# DNA-PKcs and ATM epistatically suppress DNA end resection and hyperactivation of ATR-dependent G_2_-checkpoint in S-phase irradiated cells

**DOI:** 10.1038/s41598-019-51071-6

**Published:** 2019-10-10

**Authors:** Emil Mladenov, Xiaoxiang Fan, Katja Paul-Konietzko, Aashish Soni, George Iliakis

**Affiliations:** Institute of Medical Radiation Biology, University of Duisburg-Essen Medical School, 45122 Essen, Germany

**Keywords:** DNA damage checkpoints, DNA damage response

## Abstract

We previously reported that cells exposed to low doses of ionizing radiation (IR) in the G_2_-phase of the cell cycle activate a checkpoint that is epistatically regulated by ATM and ATR operating as an integrated module. In this module, ATR interphases exclusively with the cell cycle to implement the checkpoint, mainly using CHK1. The ATM/ATR module similarly regulates DNA end-resection at low IR-doses. Strikingly, at high IR-doses, the ATM/ATR coupling relaxes and each kinase exerts independent contributions to resection and the G_2_-checkpoint. DNA-PKcs links to the ATM/ATR module and defects cause hyper-resection and hyperactivation of G_2_-checkpoint at all doses examined. Surprisingly, our present report reveals that cells irradiated in S-phase utilize a different form of wiring between DNA-PKcs/ATM/ATR: The checkpoint activated in G_2_-phase is regulated exclusively by ATR/CHK1; similarly at high and low IR-doses. DNA end-resection supports ATR-activation, but inhibition of ATR leaves resection unchanged. DNA-PKcs and ATM link now epistatically to resection and their inhibition causes hyper-resection and ATR-dependent G_2_-checkpoint hyperactivation at all IR-doses. We propose that DNA-PKcs, ATM and ATR form a modular unit to regulate DSB processing with their crosstalk distinctly organized in S- and G_2_- phase, with strong dependence on DSB load only in G_2_-phase.

## Introduction

Cellular responses to DNA damage (DDR) and particularly to DNA double strand breaks (DSBs)^1–3^ are built around ataxia-telangiectasia mutated (ATM), ATM and RAD3-related (ATR) and DNA-dependent protein kinase, catalytic subunit (DNA-PKcs)^4^, three kinases of the phosphoinositide-3-kinase (PI3K)-related family of protein kinases (PIKKs). All kinases are recruited to DSB sites by specific protein co-factors. ATM utilizes NBS1^5^, whereas ATR and DNA-PKcs utilize ATRIP^6^ and Ku80^7,8^, respectively.

DNA-PKcs, through recruitment and activation by KU heterodimer, acts as a sensor for DSBs^9^ and engages in classical non-homologous end-joining (c-NHEJ)^10,11^. ATM is the apical kinase of global cellular responses initiated by DSBs^12,13^. ATM phosphorylates the checkpoint kinase, CHK2, on multiple sites including T68^14–17^ and initiates chromatin-based DDR signaling by phosphorylating the histone variant H2AX to generate γ-H2AX^18–21^. ATM regulates DNA-end resection^22–26^ (to be referred to from now on, simply as “resection”) and promotes DSB repair by homologous recombination repair (HRR). It is also thought that ATM regulates the processing of a subset of DSBs via c-NHEJ^27^. ATR is the apical DNA replication-stress-response kinase^28^ activated through ATRIP-mediated recruitment to tracts of ssDNA coated with replication protein A (RPA)^6^. Full activation of ATR requires several factors and is complex and context dependent. ATR phosphorylates and activates CHK1^29–32^, which in S-phase inactivates the CDC25A and in G_2_–phase the CDC25C phosphatase to enforce the checkpoint response.

Despite clear functional differentiations among DNA-PKcs, ATM and ATR, their activation at the DSB and their contributions to DSB processing generate opportunities for crosstalk and functional integration. Indeed, regulatory connections between DNA-PKcs and ATM have been characterized and reviewed^4,33^. Also ATM and ATR jointly regulate resection at DSBs^24–26,34^. Furthermore, AT cells activate an ATR-dependent G_2_/M checkpoint^35,36^ and show responses suggesting crosstalk between DNA-PKcs and ATR^36^. There is also evidence that DNA-PKcs is phosphorylated by ATR *in vitro*^37^ and that it facilitates ATR-CHK1 signaling^38^. This brief outline suggests a modular integration of DNA-PKcs, ATM and ATR to sustain PIKK-signaling during DSB processing.

In a recent paper^33^, we presented experiments supporting such a modular integration of DNA-PKcs, ATM and ATR in the activation of the G_2_–checkpoint and the regulation of resection. The design of this study had two unique characteristics: First, it examined kinase crosstalk as a function of DSB load in the cellular genome, i.e. as a function of the IR-dose administered. Second, it specifically and exclusively analyzed in the G_2_-phase of the cell cycle the response of cells irradiated also in the G_2_-phase of the cell cycle^33^. These experiments showed that at low IR-doses, ATM and ATR regulate epistatically as a module the G_2_-checkpoint^33^. In this module, ATR is located at the output-node and interfaces with the cell-cycle through CHK1^33^. ATM/ATR module regulates epistatically also resection at low IR-doses. On the other hand, at high IR-doses, the modular coupling between ATM and ATR relaxes and the two kinases independently contribute to G_2_-checkpoint and resection. Notably, DNA-PKcs appears to also integrate to the ATM/ATR module and DNA-PKcs defects cause hyper-resection and G_2_-checkpoint hyperactivation^33^.

Here, we extend these studies to cells specifically exposed to IR during the S-phase of the cell cycle and analyze resection and checkpoint activation in the following G_2_-phase. Surprisingly, we discover a different regulatory organization in the outputs of the DNA-PKcs/ATM/ATR module. The results provide important insights into the cell cycle regulation of DDR that have implications for DSB processing.

## Results

### ATR fully controls the G2-checkpoint in cells sustaining DSBs in the S-phase of the cell cycle

DSBs activate the G_2_-checkpoint, which manifests as an arrest in G_2_-phase and which is experienced first by cells already in G_2_. Cells sustaining DSBs in S-phase must first complete replication and enter G_2_-phase to experience the checkpoint. The recently reported^33^ intriguing crosstalk between PIKKs in the regulation of the G_2_-checkpoint for cells irradiated in G_2_-phase, led us to inquire how this regulatory network operates when cells are irradiated in the S-phase of the cell cycle.

**Figure 1:**
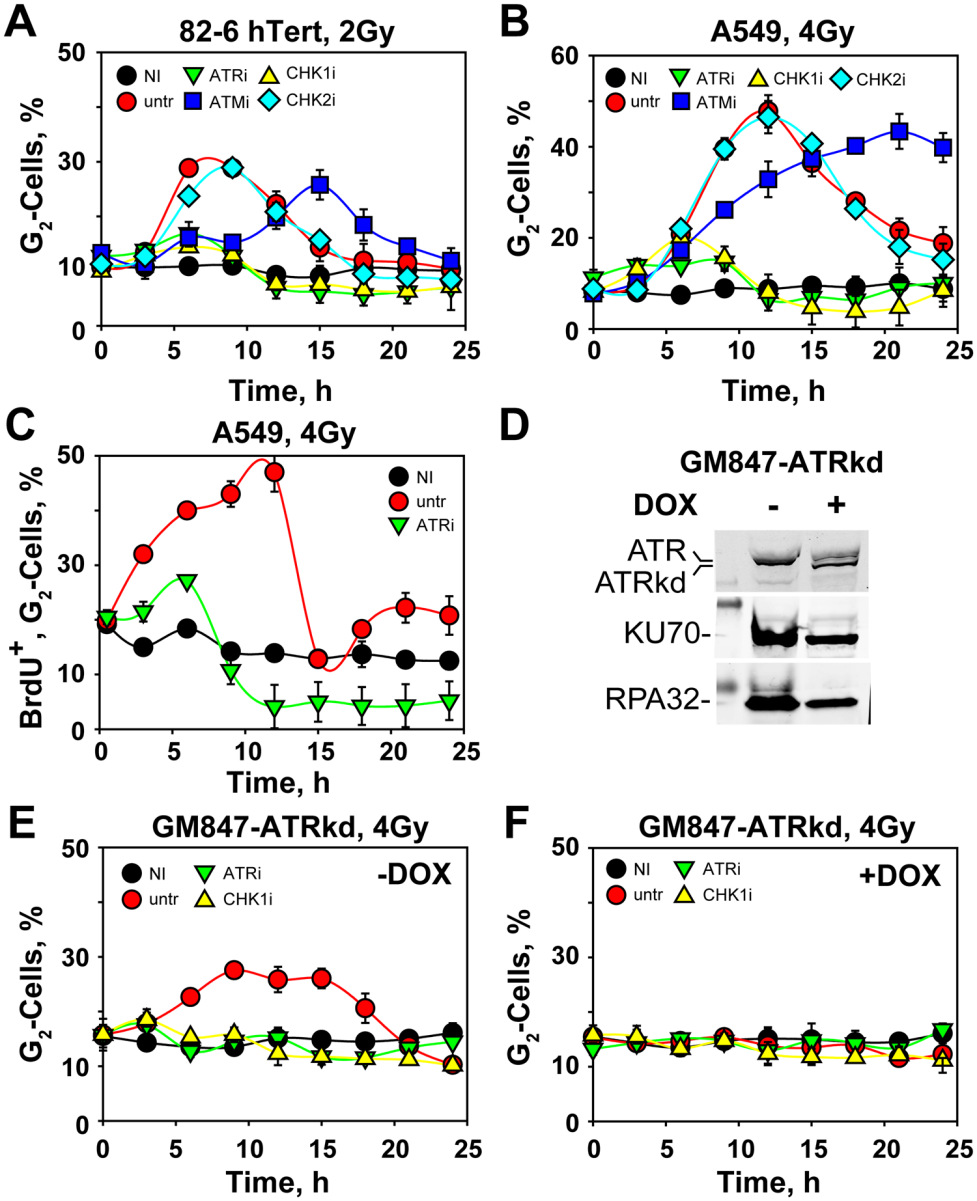
ATR is essential for the manifestation of the G_2_-checkpoint in S-phase irradiated cells. (**A**) Percentage of 82-6 hTert cells in G_2_-phase of the cell cycle as determined by single parameter flow cytometry as a function of time after exposure to 0 Gy (NI: non-irradiated) or 2 Gy of IR (X-rays). DNA content was determined by staining with propidium iodide (PI). Results are shown for untreated cells (untr), as well as for cells incubated with the indicated inhibitors. **(B)** As in (**A**) for A549 cells exposed to 4 Gy of IR. **(C)** A549 cells were pulse labeled for 30 min with BrdU and were exposed to 4 Gy of IR. The percent of BrdU positive (BrdU^+^) cells in G_2_-phase was measured as a function of time thereafter using two parameter flow cytometry. Results are shown for NI and untr cells, as well as for cells incubated with ATRi. **(D)** Western blot analysis of GM847-ATRkd cells, showing ATRkd expression after 48 h incubation with 3 µg/ml doxycycline (+DOX), or without doxycycline (-DOX). KU70 and RPA32 proteins serve as loading controls. Original, non-cropped membranes are shown in Fig. S4A. **(E)** Percentage of G_2_-phase, GM847-ATRkd cells irradiated with 4 Gy and analyzed in the absence of doxycycline (−DOX). **(F)** As in (**E**) for cells tested in the presence of doxycycline (+DOX). All data points represent the means and standard deviations (only visible when are larger than the symbol) estimated from three independent experiments.

The G_2_-checkpoint experienced by cells sustaining DNA damage in S-phase can be studied by single-parameter flow cytometry using propidium iodide (PI) staining. Exposure of actively proliferating hTert immortalized normal human fibroblasts 82-6 (82-6 hTert) to a low IR dose (2 Gy) impairs cell division without overly inhibiting ongoing DNA replication and causes a transient, time-dependent increase in the fraction of cells in G_2_–phase (Fig. 1A). This increase reflects predominantly the arrest of cells irradiated in S-phase, as only progression of cells from S-phase can increase the fraction of cells in G_2_ above the levels of non-irradiated cells. Cells irradiated in G_2_-phase, will initially remain blocked in G_2_, as they also experience an arrest^33^. Thus, checkpoint quantification for cells irradiated during S-phase is possible as increase in the G_2_ fraction over the levels of non-irradiated controls. Accumulation in G_2_ continuous in these cells for approximately 6 h, dropping subsequently and approaching pre-irradiation values 15 h post irradiation (Fig. 1A).

In order to explore the contribution of ATR in the investigated response, we have utilized a specific small molecule inhibitor, VE-821, (to be referred as ATRi) (Fig. 1A)^33^. Notably, all stages of the G_2_-checkpoint (initiation, maintenance and recovery) are abolished, as there is no evidence for notable increase in the fraction of cells in G_2_. The human lung carcinoma cell line, A549, (Fig. 1B) shows a similar response, as do also additional cell lines that are discussed below. We conclude that the G_2_-checkpoint  activated in S-phase cells exposed to low IR doses is under complete control of ATR and that this is a rather general response.

To confirm that the effects noted above specifically reflect the response of cells irradiated in S-phase, we pulse-labeled A549 cells with BrdU (10 µM, 30 min) just before IR. BrdU is incorporated into DNA and labels S-phase cells, allowing thus their specific follow-up as they progress to G_2_ by two-parameter flow cytometry (PI *vs*. BrdU) (Fig. S1A). Figure 1C shows that A549 cells, labelled and irradiated in S-phase, arrest in G_2_ with kinetics similar to those measured using only PI-staining (Fig. 1B). Notably, treatment with ATRi abrogates the G_2_-checkpoint in BrdU-labelled, S-phase A549 cells exposed to 4 Gy (Figs 1C, S1B).

To confirm the full control of ATR on the G_2_-checkpoint uncovered using small molecule inhibitors, we examined available genetic systems. GM847-ATRkd cells^39^ have integrated a Tet-On promoter controlled expression cassette, which upon administration of Doxycycline (DOX) causes the expression of an ATR fragment with inactivated kinase domain (ATRkd), (Figs 1D and S4), resulting in dominant negative inhibition of ATR activity^33^. Before expression of ATRkd, GM847-ATRkd cells irradiated in S-phase develop a G_2_-arrest that is completely abrogated by ATRi (Fig. 1E). Notably, treatment with DOX causes complete abrogation of the G_2_-arrest in cells irradiated in S-phase (Fig. 1F), and treatment with ATRi has no additional effect.

**Figure 2:**
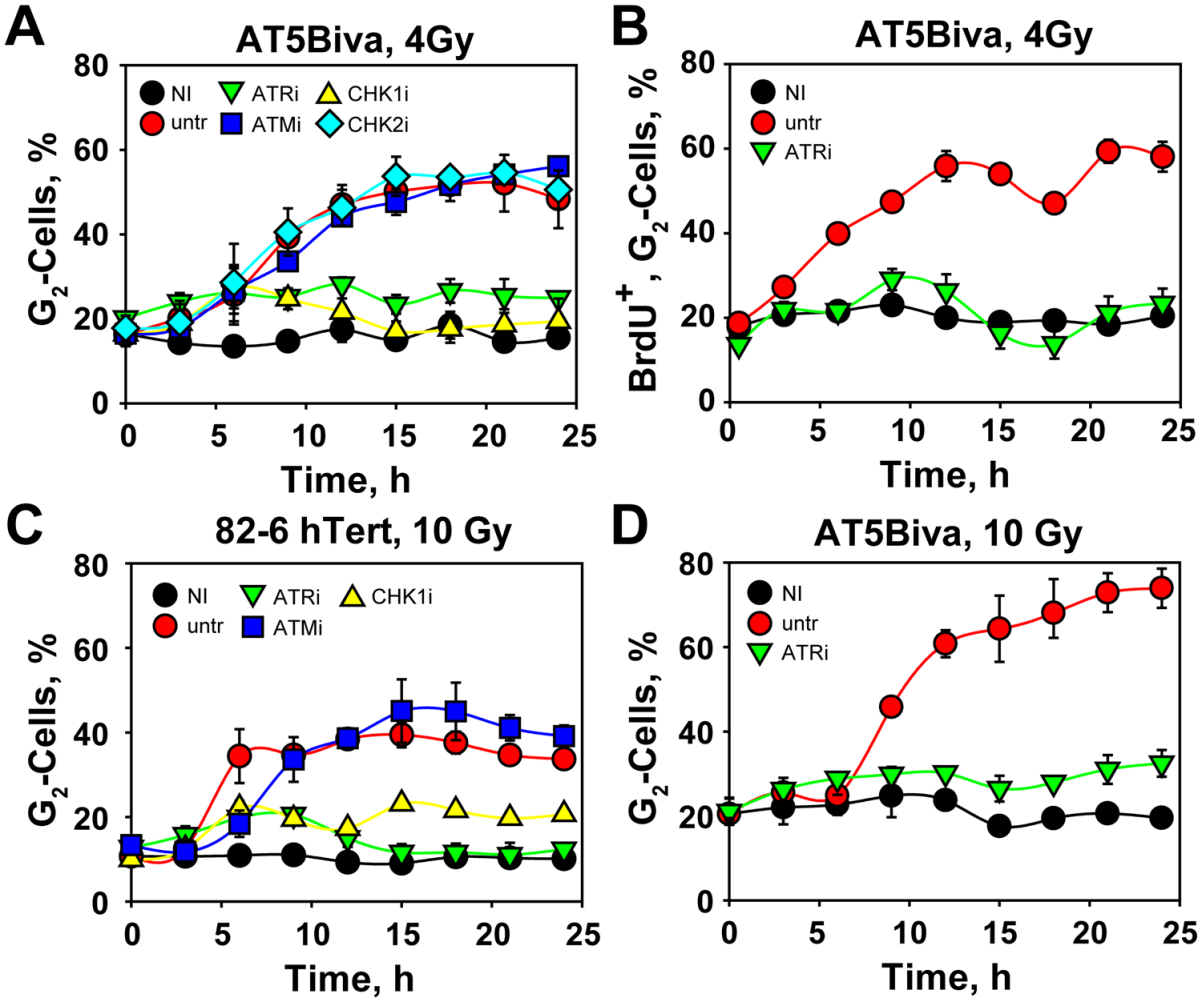
ATM deficiency results in hyperactivation of the G_2_-checkpoint when cells are irradiated in S-phase. **(A)** As in Fig. 1A for ATM deficient, AT5Biva cells exposed to 4 Gy and treated with indicated inhibitors. **(B)** As in Fig. 1C for AT5Biva cells exposed to 4 Gy and treated with indicated inhibitors. **(C)** As in Fig. 1A for 82-6 hTert cells exposed to 10 Gy and treated with the indicated inhibitors. **(D)** As in (**C**) for AT5Biva cells. All data points represent means and standard deviations (only visible when are larger than the symbol) estimated from three independent experiments.

The dominant role of ATR in the activation of the G_2_-checkpoint in cells irradiated during S-phase, raises questions regarding the contribution of a key component of the G_2_-checkpoint in cells exposed to low IR doses in G_2_-phase, the ATM protein kinase^33^. ATM deficient AT5BIVA cells irradiated in S-phase with 4 Gy, show nearly normal activation of the G_2_-checkpoint followed by a markedly prolonged arrest in G_2_ (Fig. 2A) as compared to wild-type cells (Fig. 1A). This response remains unchanged, as expected, after incubation with a specific ATM inhibitor (KU55933, to be referred to as ATMi). BrdU-labeling of AT5BIVA cells confirms that the observed effect reflects the response of S-irradiated cells (Figs 2B, S1C,D). This divergent contribution of ATM to the G_2_-checkpoint between cells irradiated in S- and G_2_-phase has generated in the past apparently contradictory results that were lively debated, until Kastan *et al*.^40^ explained these contradictions as the radically different cell cycle dependent ATM contribution shown here.

Notably, the sustained arrest in G_2_ of irradiated ATM-deficient S-phase cells depends on ATR, and is largely reversed after treatment with ATRi (Fig. 2A,B). S-phase-irradiated 82-6 hTert and A549 cells develop a very similar response after treatment with ATMi (Fig. 1A,B)^33^. An ATR contribution to this response was not considered in earlier studies^40^ but has been seen without performing a cell cycle specific analysis in our previous report^35^. The contribution of ATR to the G_2_-checkpoint has been recently investigated by others, but interpreted as contributory to ATM function and rather specific for high-LET IR-induced DNA damage^41^. Here, we show for the first time that ATR is entirely and exclusively responsible for the G_2_-checkpoint induced by low LET IR in cells exposed to low IR doses; both in a wild type, as well as in an ATM mutant genetic background.

The G_2_-checkpoint is implemented by suppressing the activity of Cdc25C, a phosphatase that activates CDK1 and drives cells into mitosis. ATM and ATR convey inhibitory signals to CDC25C through CHK2 and CHK1, respectively^4^. The dominant role noted above for ATR in the checkpoint response suggested CHK1 as the bridge to the cell cycle machinery. In line with this expectation, UCN-01, an inhibitor of CHK1 (to be referred to as CHK1i), causes a nearly complete suppression of the G_2_-checkpoint in S-phase irradiated, 82-6 hTert and A549 cells (Fig. 1A,B), as well as in non-induced GM847-ATRkd cells (Fig. 1E). A nearly complete abrogation of the G_2_-checkpoint is also observed in S-phase AT5Biva cells after treatment with CHK1i (Fig. 2A). These results demonstrate that ATR is regulating the G_2_–checkpoint in this setting by activating, practically exclusively, CHK1^42^. We note that for cells irradiated in G_2_–phase the G_2_-checkpoint only partly depends on CHK1^33^.

Since ATM inactivation strengthened/prolonged the checkpoint, we examined whether CHK2 somehow contributes to this response. Treatment of 82-6 hTert or A549 cells with a CHK2 inhibitor (Chk2 inhibitor II, BML-277, to be referred to as CHK2i) fails to generate measurable effects on the induction and recovery of the checkpoint (Fig. 1A,B), as already reported before^16,24,42–44^. The same inhibitor is also ineffective, as expected, in AT5Biva cells (Fig. 2A).

Collectively, our experiments thus far suggest that activation of the G_2_-checkpoint in cells exposed to low doses in S-phase, exclusively requires ATR that operates through CHK1 and that ATM is not contributing to checkpoint activation. On the contrary, ATM defects have in some cell lines no consequences in the activation of the checkpoint and actually often cause its prolongation. Thus, ATM suppresses in S-phase irradiated cells a G_2_-checkpoint hyperactivation that remains entirely and exclusively ATR-dependent^41,42^, and which develops independently of CHK2. This is diametrically different from the regulation established for cells irradiated in the G_2_–phase, where both ATM and ATR are equally required for G_2_-checkpoint activation^33^.

Because our previous work showed that the function of the ATM/ATR module is strongly IR-dose-dependent in G_2_–phase irradiated cells^33^, we also studied the effect of higher IR doses on the G_2_-checkpoint in S-phase irradiated cells. 82-6 hTert cells exposed to 10 Gy initiate a strong checkpoint that blocks over 40% of the cells in G_2_-phase for the period of observation (Fig. 2C). Remarkably, treatment with ATRi eliminates almost fully the checkpoint response and CHK1i treatment has a similar effect (Fig. 2C)

Exposure of AT5Biva cells to 10 Gy (Fig. 2D) also generates a substantially stronger arrest in G_2_-phase than that measured in ATM proficient cells. Strikingly, this checkpoint remains also dependent on ATR and treatment with ATRi practically abrogates the response. The same response is also confirmed in another AT cell line, AT hTert, after exposure to 10 Gy (Fig. S1E). We conclude that in contrast to G_2_–phase irradiated cells, S-phase irradiated cells show no IR-dose-dependent modulation in the regulation of the G_2_-checkpoint; which is prolonged with increasing IR dose but remains always territory of ATR. Because in G_2_–irradiated cells, DNA-PKcs affected the checkpoint in ways that resemble the effect we observe here for ATM, we inquired on the checkpoint response of DNA-PKcs deficient S-phase cells^33^.

### Defects in DNA-PKcs hyperactivate, epistatically to ATM, an ATR-dependent checkpoint in cells sustaining DSBs in S-phase

**Figure 3:**
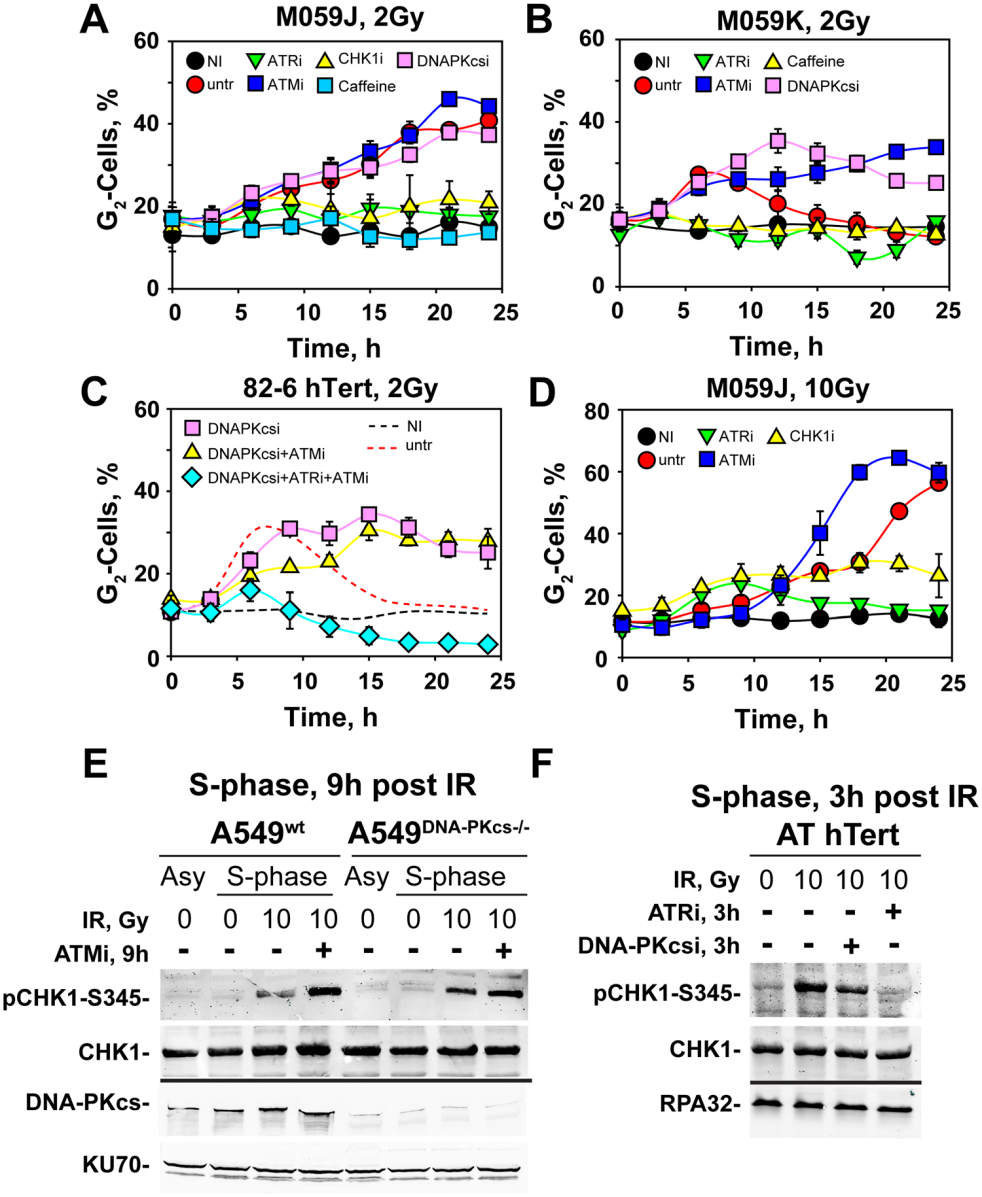
DNA-PKcs deficiency results in hyperactivation of the G_2_-checkpoint in cells irradiated during the S-phase of the cell cycle. (**A**) As in Fig. 1A for the DNA-PKcs deficient cell line, M059J, exposed to 2 Gy and treated with indicated inhibitors. **(B)** As in (**A**) for DNA-PKcs proficient M059K cells. **(C)** As in Fig. 1A for 82-6 hTert cells exposed to 2 Gy and treated with the indicated inhibitors. **(D)** As in (**A**) for M059J cells, irradiated with 10 Gy and treated with the indicated inhibitors. All data points represent the means and standard deviations (only visible when are larger than the symbol) estimated from three independent experiments. **(E)** Western blot analysis of pCHK1-S345, a marker of ATR activation, after treatment with the indicated inhibitors of parental A549 (A549^wt^) and DNA-PKcs knock-out, A549 (A549^DNA-PKcs−/−^) cells, enriched in S-phase by a single thymidine block, exposed to 10 Gy of IR and collected for analysis 9 h after irradiation. The levels of DNA-PKcs confirm the successful knockout, while the levels of CHK1 and KU70 serve as loading controls. **(F)** As in (**E**) for ATM deficient AT hTert cells analyzed 3 h after exposure to 10 Gy IR. CHK1 and RPA32 serve as loading controls. Original non-cropped images of the scanned western blot membranes are shown in Figs S4B,C.

Exposure in S-phase of DNA-PKcs deficient M059J (Fig. 3A) and HCT116 *DNA-PKcs*^−/−^ deficient cells (Fig. S2A) to 2 Gy causes a markedly stronger arrest in G_2_ as compared to M059K cells, the wild-type counterpart of M059J (Fig. 3B), or wild-type HCT116 (Fig. S2B) cells. Notably, here again, ATRi abrogates the arrest (Figs 3A and S2A), as does also CHK1i. Interestingly, caffeine, a non-specific inhibitor of ATR, causes complete suppression of the checkpoint (Fig. 3A,B, as well as S2A and S2B). When DNA-PKcs proficient 82-6 hTert (Fig. 3C) or A549 (Fig. S2C) cells are irradiated during S-phase in the presence of the specific DNA-PKcs inhibitor NU7441 (DNA-PKi)^45^, they also show prolonged activation of the G_2_-checkpoint with only slight signs of recovery in 82-6 hTert cells after 15 h. Thus, genetic ablation of DNA-PKcs and treatment with a specific DNA-PKcs inhibitor generate similar effects.

Since the hyperactivation of G_2_-checkpoint under conditions of suppressed DNA-PKcs activity is very similar to the response of ATM deficient cells, we investigated the effect of ATM inhibition in a DNA-PKcs deficient background. Strikingly, inhibition of ATM in M059J cells has no detectable additional effect on the checkpoint measured in the absence of the inhibitor and causes only a small delay in the activation of the checkpoint in HCT116 *DNA-PKcs*^−/−^ cells (Figs 3A and S2A). Also, in DNA-PKcs proficient 82-6 hTert (Fig. 3C) cells, combined treatment with DNA-PKcsi and ATMi generates a response similar to single inhibitor treatment. Under all above described conditions, the hyperactivated G_2_-checkpoint relies almost fully and exclusively on ATR and CHK1 and is abrogated by ATRi or CHK1i (Figs 3A and S2C). Notably, in 82-6 hTert or A549 cells, combined treatment with DNA-PKcsi + ATMi + ATRi practically eliminates the checkpoint response (Figs 3C and S2C).

The practically complete dependence on ATRi and CHK1i of the checkpoint activated in G_2_ in S-irradiated DNA-PKcs deficient cells is also observed after exposure to 10 Gy, although here treatment with ATMi causes a slightly earlier initiation of the G_2_-checkpoint (Figs 3D and S2D). Collectively, the results of this section demonstrate that DNA-PKcs exerts pronounced inputs into the G_2_-checkpoint, subject to relatively small quantitative but not qualitative adjustments with IR-dose. Thus, DNA-PKcs activity is required to suppress checkpoint hyperactivation, or, in an alternative view, checkpoint recovery in the range of doses examined. In this function, DNA-PKcs operates epistatically with ATM, suggesting that in S-phase irradiated cells the two kinases function as a module to regulate processes that prevent the hyperactivation (or recovery) of an ATR-dependent checkpoint.

To directly demonstrate hyperactivation of ATR signaling under conditions of DNA-PKcs and ATM deficiency, we analyzed phosphorylation of CHK1 at Serine 345 (pCHK1-S345). This event is ATR-specific and is commonly used as surrogate marker of ATR activation. To generate results relevant to S-phase irradiated cells we employed chemical synchronization protocols that generate populations enriched in S-phase cells, which we exposed to IR. We selected A549 cells for these experiments because we had synchronization protocols, as well as a DNA-PKcs knockout mutant generated using CRISPR/Cas9 technology. Figure S2E (upper panels) shows the cell cycle distribution of irradiated (10 Gy) S-phase-enriched cells, while the lower panels their distribution 9 h later, for wild-type (left panels) and DNA-PKcs deficient (right panels) cells. Analysis of pCHK1-S345 in wild-type cells (Fig. 3E, left lanes) shows the expected increase in signal documenting ATR activation that is further potentiated by inhibition of ATM. Formation of pCHK1-S345 is enhanced in DNA-PKcs-deficient A549 cells and this effect is further amplified by inhibition of ATM (Fig. 3E, right lanes). Figure S2F shows S-phase enriched populations generated in AT hTert cells and exposed to 10 Gy. Analysis carried out 3 h later when a significant proportion of cells reach G_2_-phase shows strong production of pCHK1-S345 that is almost completely abrogated following treatment with ATRi, while it remains robust after treatment with DNA-PKcsi (Fig. 3F). Quantitative comparisons between the results obtained with different cell lines is confounded by differences in the initial distribution throughout the cell cycle and the subsequent progression to G_2_-phase of the irradiated cells. Despite this limitation, the results demonstrate ATR hyperactivation under conditions of ATM and DNA-PKcs deficiency.

The mechanistic reliance of the G_2_-checkpoint on ATR in S-phase irradiated cells, places at the forefront the mechanism of ATR activation which has resection at its center. Therefore, in the following sections, we analyze in detail this endpoint under conditions similar to those employed in the checkpoint experiments.

### ATR exerts limited control on G_2_-phase-resection in S-phase irradiated cells

**Figure 4:**
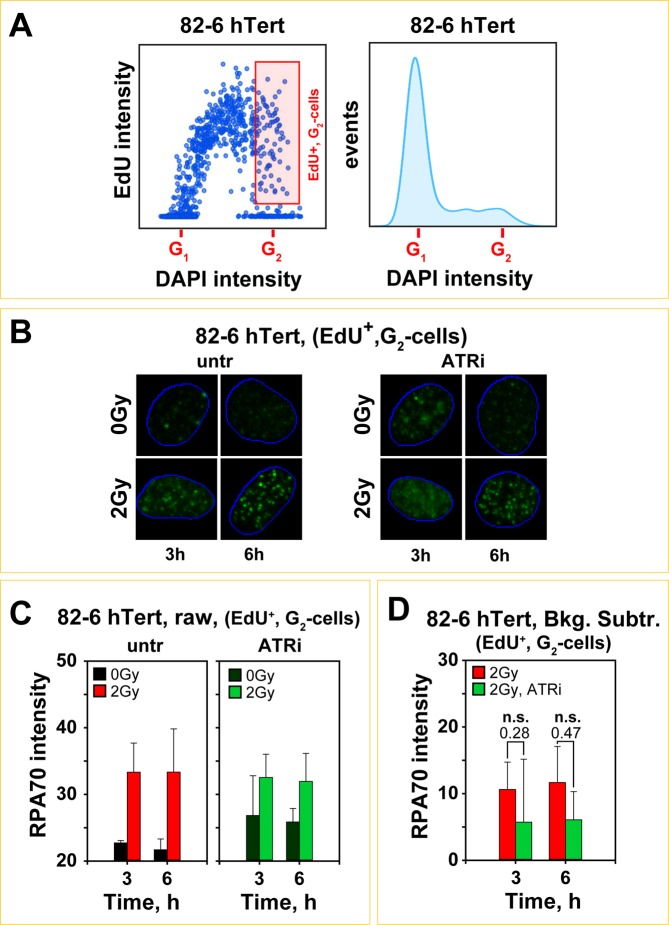
ATR plays no role in the regulation of DNA end-resection when cells are exposed to low doses of IR in S-phase of the cell cycle. (**A**) Automated high-throughput IF image analysis (Metasystems), showing representative dot plots of EdU *vs* DAPI signals obtained by scoring of approximately 1600 exponentially growing 82-6 hTert cells (left panel). Gate for selecting EdU positive (EdU^+^), G_2_-phase cells to analyze resection by quantification of RPA70 total signal intensity, is shown by the red rectangle. Right panel illustrates the cell cycle distribution of the analyzed cell population derived by the intensity of the DAPI signal. **(B)** Representative images showing RPA70 signal, a measure for DNA end-resection at DSBs, in EdU^+^, G_2_-phase 82-6 hTert cells, 3 and 6 h after exposure to 2 Gy in the absence or presence of ATRi. The blue contours indicate the location of the nucleus, after counterstaining of DNA with DAPI. **(C)** Quantitative analysis of total RPA70 signal intensity in EdU^+^, G_2_-82-6 hTert cells at 3 and 6 h after exposure to 2 Gy in the presence or absence of ATRi. The raw RPA70 signal in non-irradiated and irradiated cells, treated or not with ATRi, are plotted. **(D)** Background subtracted quantitative analysis of results plotted at (**C**). Data points represent the mean and standard deviation calculated from three independent experiments. A student t-test was used for statistical analysis and the individual p-values are indicated.

We employed immunofluorescence (IF) to evaluate DNA end-resection by measuring RPA70 (the largest subunit of the RPA complex) retention at chromatin as a function of time after irradiation^33^. For a specific analysis in G_2_-phase for cells irradiated in the S-phase, we labeled cells with EdU (30 min) before IR exposure and quantitated RPA70 signal in EdU positive (EdU^+^), G_2_-phase cells, identified by the intensity of the DAPI-signal (Fig. 4A). This cohort reflects cells that were in S-phase at the time of irradiation and which have completed replication and entered G_2_-phase in the post-irradiation incubation time interval. Figure 4A outlines this form of analysis and the gates adopted to measure RPA70 signal in such cells (gate marked in red). Figure 4B shows representative IF images of the analyzed population of 82-6 hTert cells, treated or not with ATRi, exposed to 0 or 2 Gy and processed at different times thereafter. Figure 4C shows quantification at the indicated times of the integral RPA70 signal in this cohort of cells (100–150 EdU^+^, G_2_-phase cells analyzed for each data point). Results of irradiated cells are plotted together with results obtained in an identical manner from non-irradiated cells treated similarly and measured within the same gates (background signal) (Fig. 4C). We use integral RPA signal intensity per cell as a parameter in the IF analysis, arguing that it reflects the level of ATR activation better than scoring of individual foci^33^. In addition, it allows comparison with integral signal analysis carried out to measure resection using flow cytometry (see below).

It is evident (Fig. 4C,D) that at 3 and 6 h after exposure to IR a significant increase in RPA70 signal over background is observed in EdU^+^, G_2_-cells, suggesting resection at DSBs that sustains the G_2_-checkpoint (Fig. 1A). ATRi treatment leaves in irradiated cells RPA70 signal practically unchanged (Fig. 4C). Notably, in non-irradiated cells treated with ATRi, RPA70 signal is markedly elevated (Fig. 4C). This increase likely reflects binding of RPA complex to ssDNA persisting in cells from the S-phase that have entered G_2_-phase; it may be generated as a result of problems encountered during DNA replication and which are enhanced after treatment with ATRi. Indeed, it is known that even under normal replication conditions, late replicating loci in heterochromatin and loci with fragile sites and repetitive elements, suffer replication fork stalling^46^ and may complete replication in G_2_–phase^47,48^. Such effects are exaggerated after treatment with ATRi^49,50^ and likely cause the increase in RPA70 signal observed in non-irradiated cells. If we consider this increased signal as the “legitimate” background of the corresponding irradiated samples and subtract it, the net RPA70 signal increase shown in Fig. 4D is obtained. Although these results appear to show a signal reduction in ATRi treated cells after IR exposure, the effect fails to reach statistical significance.

**Figure 5:**
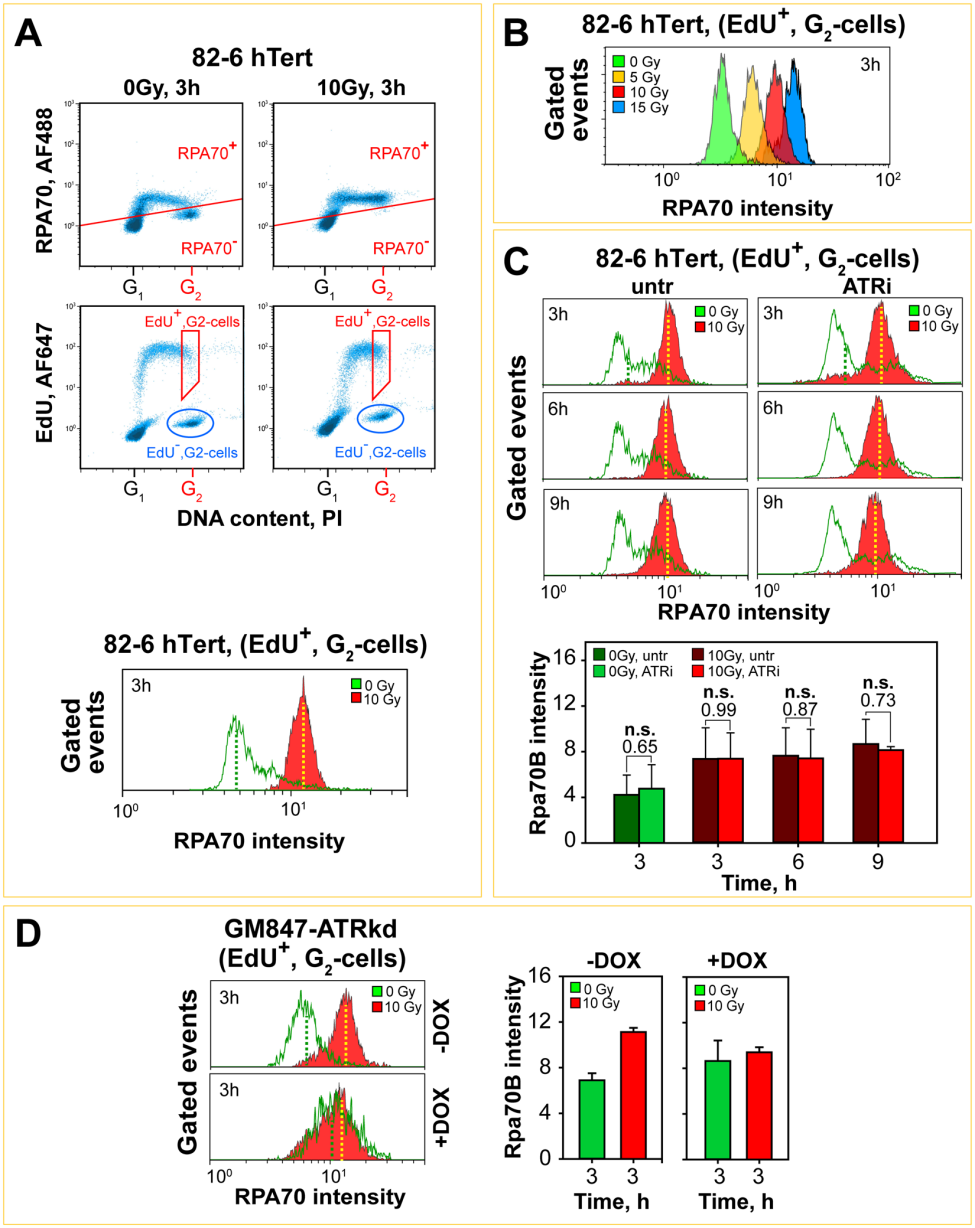
ATR plays no role in the regulation of DNA end-resection in cells irradiated with high IR doses during S-phase when analyzed in the subsequent G_2_-phase of the cell cycle. (**A**) Summary of the three-parametric flow cytometry analysis utilized to quantitate DNA end-resection in cells exposed to high IR doses in S-phase. Plots illustrating RPA70 *vs*. PI signals (upper panels), or EdU *vs*. PI signals (middle panels). On the middle panels the gates applied to quantify DNA end resection in G_2_-cells irradiated in S-phase (EdU^+^) are also indicated. The lower part of the figure shows histograms of RPA70 signal evaluated in non-irradiated (0 Gy, green) or irradiated (10 Gy, red) EdU^+^, G_2_-phase cells. **(B)** Histograms of RPA70 signal intensity measured in EdU^+^, G_2_–phase, 82-6 hTert cells exposed to 5, 10 and 15 Gy. **(C)** Upper panels: Histograms of the intensity of RPA70 signal as a function of time in EdU^+^, G_2_-phase, 82-6 hTert cells irradiated with 10 Gy in the presence or absence of ATR inhibitor. Lower panels: Quantitative analysis of three independent experiments, showing the arithmetic means for RPA70 signal intensity. The error bars represent standard deviations. A student t-test was utilized for statistical analysis. The individual p-values are indicated on top. **(D)** Left panel: Histograms showing the RPA70 intensity, 3 h after exposure of GM847-ATRkd cells to 10 Gy after pretreatment (+DOX) or not (−DOX) with doxycycline. Right panel: Quantitative analysis of two independent experiments, showing the arithmetic means of RPA70 intensity. The error bars represents standard deviations.

To study resection at higher IR doses, we employed a quantitative flow cytometry-based method^33,51^. Cells are incubated, with EdU to label cells in S-phase and resection is measured by detecting RPA70 in EdU^+^, G_2_-phase cells, identified by co-staining of DNA with propidium iodide (PI). The upper panels in Fig. 5A show as an example raw data as dot plots and the gates used to quantitate RPA, EdU and PI signals using results obtained 3 h after irradiation of 82-6 hTert cells with 0 or 10 Gy. The histograms in the lower panel of Fig. 5A show intensity distribution of RPA70 signal in the defined gates in irradiated and non-irradiated cells. The robust RPA70 signal increase observed in cells exposed to 10 Gy indicates extensive resection at DSBs. Figure 5B shows that IR-induced resection can be conveniently quantitated in a range of doses between 5 and 15 Gy using this method.

We employed the above protocols to analyze resection as a function of time after exposure of 82-6 hTert cells to 10 Gy. The results in Fig. 5C show robust resection at 3 h, 6 h and 9 h after IR, in line with the activation of ATR required to sustain the checkpoint. Notably, and in line with the IF analyzes, inhibition of ATR has little effect on the overall IR-induced resection (Fig. 5C, upper panels). However, here again slight increase in RPA70 signal is measured in non-irradiated cells. The lower panels in the Fig. 5C summarize the results of three experiments using as parameter the arithmetic mean of RPA70 signal distribution. Here again, ATRi has no effect on net signal intensity, while non-irradiated controls show higher background signal. Plotting of the same results using the median of RPA70 signal distribution as a parameter instead, leads to similar conclusions (Fig. S3A).

A similar analysis in non-induced GM847-ATRkd cells exposed to 10 Gy also shows robust resection at 3 h in G_2_ for S-phase irradiated cells (EdU^+^) (Fig. 5D, left panel). Inhibition of ATR by administration of DOX causes no detectable suppression of IR-induced resection but causes also a marked increase in the RPA70 signal in non-irradiated cells (Fig. 5D, left panel). The bar graphs in Fig. 5D (right panels) show the compiled results of two experiments. Similar trends are also observed in A549 cells, although the double peaks in the RPA70 signal distributions, particularly in the non-irradiated samples, complicate the quantitative analysis of the results (Fig. S3B).

Collectively, the above results show robust resection in G_2_–phase in cells irradiated during the S-phase of the cell cycle, in line with the ATR dependent checkpoint documented in the previous section. ATRi treatment failed to change the IR-induced RPA70 signal, but increased the background signal in non-irradiated cells. As a result the background-corrected net increase in RPA70 signal in irradiated, ATRi treated cells could be interpreted as showing partial suppression of resection by ATRi. While final answer for this ambiguity will require alternative methods of resection analysis in cells irradiated in S-phase (see below), we suggest that for cells irradiated in S-phase, ATR exploits resection for activation and implementation of the G_2_-checkpoint, but that it exerts itself only limited regulation on resection. This is in contrast to the strong regulatory inputs ATR has in cells irradiated in G_2_–phase, where ATR inhibition suppresses resection: completely at low and partially at high IR-doses^33^.

**Figure 6:**
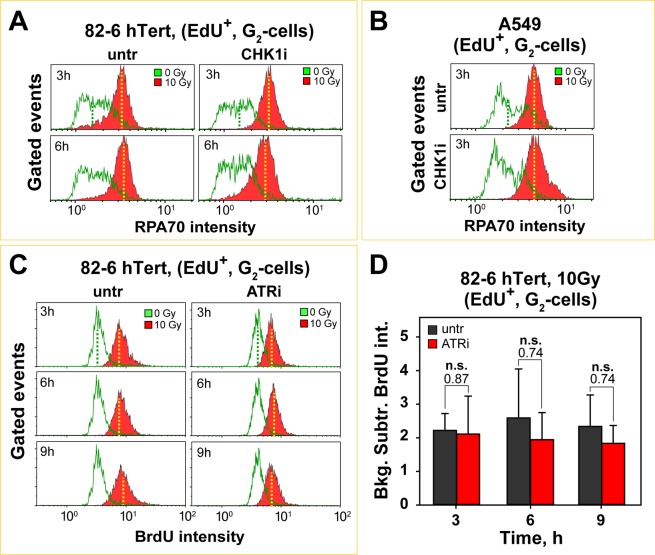
ATR and CHK1 are not essential for the regulation of DNA end-resection in cells irradiated with high IR doses. (**A**) Histograms of RPA70 intensity, measured in EdU^+^, G_2_-cells, 3 and 6 h after exposure of 82-6 hTert cells to 10 Gy in the presence or absence of CHK1i. **(B)** Same as in **(A)** for A549 cells, collected 3 h after exposure to 10 Gy. **(C)** Histograms of BrdU signal intensity as a function of time after exposure of 82-6 hTert cells to 10 Gy of IR in the presence or not of ATRi. BrdU intensity is measured in EdU^+^, G_2_-phase cells, gated according to the regions illustrated in Fig. 5A. **(D)** Quantitative analysis of BrdU signal intensity at indicated times after IR. The results show values calculated after subtracting the BrdU signal measured in non-irradiated cells from that measured in irradiated cells. The data represents averages from 3 independent experiments and the error bars show the standard deviation. A student t-test was utilized for statistical analysis and the calculated p-values are indicated.

Inhibition of CHK1 leaves G_2_-phase resection unchanged in S-phase irradiated 82-6 hTert and A549 cells (Fig. 6A,B). As for ATRi, albeit less pronounced, CHK1i increases RPA70 signal intensity in non-irradiated cells generating the same issues in the analysis of CHK1i effect on resection as discussed above for ATRi. Since CHK1i failed to suppress resection in G_2_-irradiated cells^33^, we infer that the present set of data also suggests no effect of CHK1 on IR-induced resection. If this is indeed true it means that the effect of ATR/CHK1 inhibitors on non-irradiated cells may not be fully transmitted to irradiated cells. We present next results that further support this notion.

To validate the above effects on resection with an independent method, we grew 82-6 hTert cells for 24 h in the presence of BrdU, labeled them with EdU for 30 min and exposed them to IR. At different times after IR, we analyzed cells by flow cytometry to measure, in a cell cycle dependent manner, ssDNA by staining non-denatured DNA with BrdU-specific antibody. The overall analysis is very similar to that described for RPA, except that BrdU signal on ssDNA is instead detected to assess resection. Figure 6C (left panels) shows that exposure to 10 Gy robustly increases BrdU signal in EdU^+^ cells analyzed in G_2_-phase at 3, 6, and 9 h post-irradiation, confirming robust resection that sustains the checkpoint. Treatment with ATRi leaves here again the overall BrdU signal intensity practically unchanged (Fig. 6C, right panels). Non-irradiated samples show a smaller increase in BrdU signal after treatment with ATRi as compared to the RPA70 measurements presented above. Furthermore, background subtracted analysis of such distributions from three experiments (Fig. 6D) supports the notion that ATRi does not affect resection in S-phase irradiated cells. Similar conclusions are drawn when the results are analyzed using the median of the BrdU signal distribution instead (Fig. S3C).

### DNA-PKcs and ATM defects lead to persistent resection in an epistatic manner in subsequent G_2_-phase in S-phase irradiated cells

**Figure 7:**
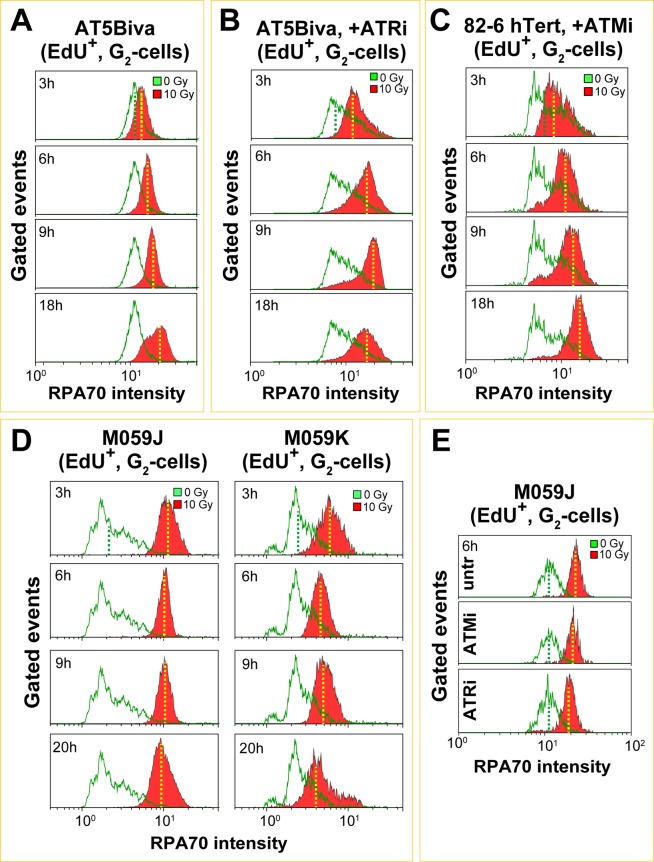
ATM and DNA-PKcs deficiency cause hyperresection in cells exposed to high IR doses in S-phase and analyzed in the subsequent G_2_-phase of the cell cycle. (**A**,**B)** RPA70 intensity, measured in EdU^+^, G_2_-phase, AT5Biva cells, treated or not with ATRi and exposed to 10 Gy of IR. Other details as in Fig. 5C. **(C)** RPA70 intensity, measured in EdU^+^, G_2_-phase, 82-6 hTert cells treated with ATMi. **(D)** RPA70 intensity, in EdU^+^, G_2_-phase, M059J and M059K cells, exposed to 10 Gy of IR and collected at the indicated times after irradiation. **(D)** RPA70 signal intensity in M059J cells treated with the indicated PIKK inhibitors.

When AT5BIVA cells are irradiated in S-phase, resection measured in G_2_–phase is overall reduced as compared to ATM proficient cells, but persistent over time (Fig. 7A) explaining the persistent checkpoint developing under these conditions (Figs 2 and S1E). Treatment of these cells with ATRi has only a small effect on resection (Fig. 7B). Notably, ATM deficiency partly rescues the effect of ATRi on DNA replication in non-irradiated cells, mitigating thus some of the above outlined complications in the interpretation of the results obtained. Similar trends are also seen in AT hTert cells, before and after treatment with ATRi (Fig. S3D). When ATM proficient, 82-6 hTert cells are treated with ATMi, marked and persistent resection develops (Fig. 7C). Thus, in S-phase irradiated cells, ATM suppresses late hyperresection.

DNA-PKcs deficient M059J cells exposed to 10 Gy in the S-phase show in G_2_-phase enhanced resection that persists for longer times (Fig. 7D, left panels) as compared to M059K cells (Fig. 7D, right panels). Treatment of these cells with ATMi fails to significantly increase resection, suggesting that for this endpoint and under the conditions employed ATM and DNA-PKcs work epistatically. ATRi exerts no effect on resection in this genetic background as well, and in this particular cell line it only has a relatively small effect on RPA70 signal intensity in non-irradiated cells (Fig. 7E). Similar conclusions can be drawn from results obtained in DNA-PKcs deficient, HCT116 cells (Fig. S3E). Also, treatment with CHK1i in a DNA-PKcs deficient background does not affect the DNA end resection (Fig. S3F). Thus, for cells irradiated in S-phase, both ATM and DNA-PKcs function in an epistatic manner to suppress resection at DSBs during their processing in G_2_–phase, and this response mirrors closely their effects on the G_2_-checkpoint.

## Discussion

### ATR exclusively activates the G_2_-checkpoint in cells irradiated in S-phase

**Figure 8:**
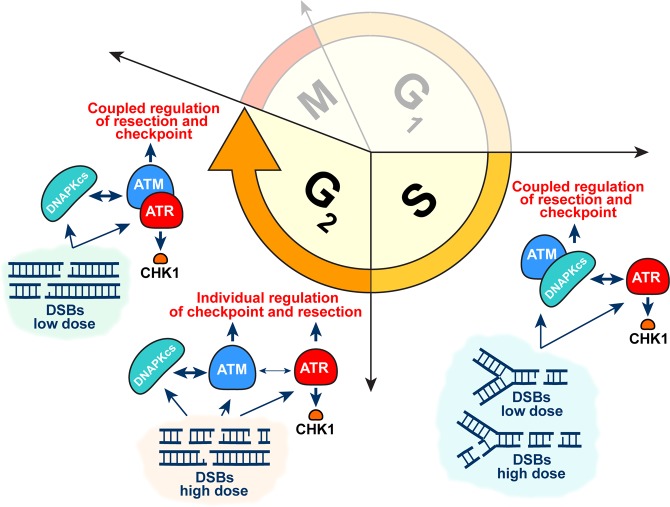
Schematic representation of a model outlining the postulated crosstalk between the members of PIKK kinase family (DNA-PKcs, ATM and ATR) in regulation of the G_2_-checkpoint and DNA end-resection. Shown are presumed changes in this crosstalk occurring as a function of IR dose in cells irradiated in G_2_-phase of the cell cycle as described before^33^, as well as the altered crosstalk between these kinases in the regulation of the same endpoints when cells are irradiated in the S-phase, as described in the present paper. See Discussion for details.

While the individual functions of ATM and ATR are relatively well-characterized^4^, aspects of their functional cooperation and crosstalk continue to emerge^33^. We recently reported intriguing functional interactions between ATM and ATR in the regulation of the G_2_-checkpoint in cells irradiated in G_2_-phase, showing striking mechanistic adaptations with increasing load of DSBs in the genome^33^. Specifically, we discovered a complete functional coupling between ATM and ATR in the regulation of the G_2_-checkpoint in cells with low numbers of DSBs induced by exposure to low doses of IR (see model description in Fig. 8). Under these conditions and phase of the cell cycle, ATM and ATR are equally required for the activation of the checkpoint, and inhibition of either kinase completely abrogates this activation^33^. We use here the term epistasis to describe this relationship, but we caution the reader that there are alternative definitions of this term^52^. This previously unreported functional coupling between ATM and ATR appears directional, connecting to the cell cycle machinery primarily through ATR to CHK1 signaling (Fig. 8).

Strikingly, this tight modular interdependence relaxes when cells are exposed to high IR doses. Now, independent outputs from ATM and ATR activate the G_2_-checkpoint and inhibition of both kinases is required to fully suppress this activation (Fig. 8). Either way, G_2_-checkpoint activation remains the exclusive territory of the ATM/ATR duo. This dose-dependent modular integration between ATM and ATR raises ATR relevance to the same level as ATM, and suggests that views of an ATM-centered regulatory organization of the G_2_-checkpoint^53^ need revision.

The results presented here, while confirming^33^ a modular cooperation between DNA-PKcs, ATM and ATR in the overall regulation of the G_2_-checkpoint, they also uncover striking and unexpected differences in the specific wiring of the module when cells are irradiated in S-phase. Thus, in stark contrast to the epistatic and dose-dependent regulation of the checkpoint by both ATM and ATR in cells irradiated in G_2_-phase, in cells irradiated in S-phase the G_2_-checkpoint depends entirely on ATR that functions practically exclusively through CHK1; moreover, the mechanism of this regulation is not fundamentally altered with increasing IR-dose (see model description in Fig. 8).

This observation is highly significant mechanistically, as it suggests that with the transition of a cell from S- to G_2_-phase, regulatory and possibly also structural changes in genome organization occur that profoundly modify the mechanistic underpinnings of the G_2_-checkpoint. It is evident therefore, that only strictly cell cycle phase-specific analysis of DDR will generate “pure” results for sound mechanistic interpretations advancing our understanding of its molecular underpinnings. When the experimental data “mix” the diametrically different responses of cells irradiated in S- and G_2_-phase, misleading conclusions are likely to be drawn, or apparently contradictory results may be obtained in different cell systems.

### In cells irradiated in S-phase, ATM and DNA-PKcs epistatically suppress G_2_-checkpoint hyperactivation

Not only is in cells irradiated in the S-phase ATR the sole controller of the G_2_-checkpoint, but in addition ATM, the intimate partner of ATR for cells irradiated in G_2_-phase, assumes now a diametrically different contribution to the checkpoint. In this setting, ATM is not required in any way for the activation of the checkpoint, but supports instead its recovery; as a consequence ATM deficiency causes G_2_-checkpoint prolongation and hyperactivation. This is similar to the function of DNA-PKcs in cells irradiated in G_2_-phase^33^. Strikingly, in cells irradiated in S-phase, the functions of ATM and DNA-PKcs are epistatic in the sense that inhibition of either kinase generates equivalent effects and inhibition of both kinases produces no additivity. We conclude that both kinases epistatically regulate the same process related to the recovery from the checkpoint, and that defects cause ATR and checkpoint hyperactivation (Fig. 8).

### Reduced control of resection by ATR in G_2_-phase in cells irradiated in S-phase

Resection at a DSBs is an important determinant of DSB-repair pathway choice favoring HRR, SSA, and alt-EJ^10,54,55^. The fundamental requirement for resection in ATR activation links thus a key aspect of checkpoint control to the specific forms of DSB processing that require resection^56^. Indeed, c-NHEJ is not linked to ATR signaling and the G_2_-checkpoint^57–60^; an observation fully in line with c-NHEJ’s fast kinetics. The fact that resection accompanies the activation of the G_2_-checkpoint in cells irradiated in S-phase provides mechanistic explanation for the involvement of ATR. Strikingly though, in contrast to G_2_-irradiated cells, where ATR is not only passively responding to resection, but is also actively regulating it^33^, in cells irradiated in S-phase ATR only responds to resection but has only limited, if any, control in the process. As for cells irradiated in G_2_, CHK1 is not playing a role in the regulation of resection. This immediately raises the question as to whether the ATR mediated regulatory phosphorylation of CtIP^61^ occurs differently in cells irradiated in S-phase versus cells irradiated in the G_2_-phase of the cell cycle^56^.

We are aware that the resection measurements in S-phase-irradiated cells have some ambiguity, as a consequence of ATRi effects on DNA replication that increase RPA signal in non-irradiated S-phase cells when they reach G_2_-phase. However, the lack of any notable reduction in the RPA70 signal in the irradiated population by two independent methods is in line with this conclusion.

### DNA-PKcs and ATM function in an epistatic manner to suppress hyperresection

The last significant contribution of the results reported here is the epistatic suppression of hyperresection by DNA-PKcs and ATM in cells irradiated in S-phase when they reach G_2_-phase. This persistent and epistatic hyperresection observed in the absence of DNA-PKcs and ATM activities explains the above discussed hyperactivation of the checkpoint as hyperactivation of ATR. These results tightly link resection to ATR activation and the mounting of ATR-dependent checkpoints (Fig. 8). There is evidence for links between DNA-PKcs and ATM with the former exerting strong negative regulation on ATM through phosphorylation at multiple sites^62^. DNA-PK also stimulates the MRN complex and CtIP for efficient endonucleolytic processing of DNA ends under physiological conditions^63^. How these interesting observations feed into the cell cycle dependent regulation in the crosstalk between members of the PIKK kinase family (DNA-PKcs, ATM and ATR) will be subject of future investigations.

However, whereas DNA-PKcs deficiency causes increased levels of resection that persist, ATM deficiency leads to reduced initial levels of resection that also persist. This points to qualitatively similar inputs between DNA-PKcs and ATM in the regulation of resection and G_2_-checkpoint activation that have different quantitative manifestations.

### A model integrating the activities DNA-PKcs, ATM and ATR in S- and G_2_-phase

In aggregate, we describe cell cycle specific contributions of PIKK family members (DNA-PKcs, ATM and ATR) to the regulation of G_2_-checkpoint and DNA end-resection that are consistent with crosstalk and possibly cross-regulation among these kinases^33^. Therefore, we postulate their modular integration. Our working hypothesis is summarized in the model outlined in Fig. 8. The first opportunity of functional integration among these kinases derives from their ability to be recruited to DSBs at different stages in their processing, both for cells irradiated in S- and in G_2_-phase. Indeed, for DSBs that will eventually undergo resection, all three kinases will be successively recruited, and will contribute, in varying ways as we saw above, to the regulation of this resection process. The modular integration model proposed here, specifically applies to this subset of DSBs and it is also this subset of DSBs, which according to our results, has links to the checkpoint response.

While the initial recognition and binding to DSBs will likely start from DNA-PK, recruitment and activation of ATM and subsequently of ATR will ensue. Such sequential functional integration for the processing of an individual DSB explains why the substrates of DNA-PKcs, ATM and ATR are largely overlapping: to ensure sustained DDR signaling as DSBs transit through the different processing steps.

While this modular integration in the functions of DNA-PKcs/ATM/ATR ensure optimized processing of DSBs (acting as a form of optimization module), it is functionally important that recruitment of anyone of these kinases to the DSB can also occur independently of the presence of the other kinases, explaining why frequently deletion of one kinase does not eliminate the activation of the others. Thus, the function of DNA-PKcs in c-NHEJ does not require ATM or ATR, and ATM is fully activated in the absence of ATR activity.

Furthermore, our results suggest coupled functions for essential aspects of DDR such as the regulation of checkpoint activation and recovery, as well as the regulation of resection. Our results indeed point to a tight linkage between the former and the latter as already reported for the yeast^56^. Notably, we could demonstrate that these coupled functions are wired in the different manner in cells sustaining DSBs in S-phase and the G_2_-phase of the cell cycle (Fig. 8). Thus, in cells sustaining DSBs in G_2_-phase, ATM and ATR function as a tightly and epistatically operating pair that has under its full control resection and G_2_-checkpoint activation. Functional uncoupling requires high loads of DSBs in the genome and may reflect a means cells employ to better cope with high loads of DSBs. In this sub-module, ATR occupies the exit node with reference to cell cycle regulation, mediated by Chk1 and possibly an additional unidentified kinase. Here, the third member of the family, DNA-PKcs, regulates resection and checkpoint recovery (Fig. 8).

On the other hand, in cells irradiated in S-phase the checkpoint is regulated entirely by ATR that is activated by resection, controlled by DNA-PKcs/ATM that now operate as a pair epistatically. The exact role of ATR in the regulation of this resection will require additional investigations. Connections between resection and checkpoint affecting recovery from the checkpoint and a defining role of KU in this response have been reported for *Saccharomyces* cells after inducing a single DSB^56^. The characterization of the molecular underpinnings of DNA-PKcs, ATM/ATR interactions is a promising area for future mechanistic investigations.

## Methods

### Cell culture and irradiation

All cell lines employed^33^ were grown in 10–20% fetal bovine serum (FBS)-supplemented cell culture media, at 37 °C in an atmosphere of 5% CO_2_ in air. DNA-PKcs knock-out and parental A549 cell lines, parental HCT116 and DNA-PKcs knock-out HCT116 cells were maintained in McCoy’s 5 A medium; ATM deficient cell lines, AT5BIVA and AT hTert (GM2052), as well as the conditionally ATR-deficient, GM847-ATRkd, and 82-6 hTert cells, were grown in minimum essential medium (MEM), supplemented with 1% non-essential amino acids. DNA-PKcs deficient, M059J, and their wild-type counterparts, M059K, cells were maintained in Dulbecco’s modified Eagle’s medium (D-MEM). If not stated otherwise, cells were exposed to IR at 37 °C using a 320 kV X-ray machine with a 1.65 Al filter (GE-Healthcare). The dose rate at 500 mm distance from the source was 2.7 Gy/min.

### Synchronization of cells in S-phase

In order to enrich cells in S-phase of the cell cycle a single thymidine block was applied. Cells were treated with 2 mM thymidine for 18 h and were released by single washing with PBS and immediate incubation in fresh growth medium. Cells were allowed to progress through the S-phase for 3 h and were treated with PIKK inhibitors 1 h prior to irradiation. Cells were collected at 3 or 9 h after irradiation.

### Generation of DNA-PKcs knock-out A549 cell line

CRISPR/Cas9 technology was utilized for the generation of DNA-PKcs knock out cells. For this purpose, A549 cells (A549-wt) were transfected with Cas9 expression plasmids, which were constructed to express a DNA-PKcs exon specific gRNA. The plasmid also co-expresses a GFP protein, which was utilized to select transfected cells by cell sorting for further analysis. Individual A549 clones were isolated and DNA-PKcs expression was monitored by western blottting (Fig. 3E). The isolated DNA-PKcs negative clones were further confirmed by analyzing their sensitivity to IR using clonogenic assays (data not shown). A pool of the following qRNAs was applied to generate DNA-PKcs knockout, A549 cells; gRNA1-AAAGGCATCAACTCAGGGAC, gRNA2- CAGCAAGTGCACCTGTGTAG, gRNA3- ATCGACTTTGGGCATGCGTT, gRNA4- GATCACGCCGCCAGTCTCCA, gRNA5- CAGACATCTGAACAACTTTA.

### Treatment of cells with kinase inhibitors

Caffeine (Sigma-Aldrich) was dissolved in distilled water at 100 mM and was used at a final concentration of 4 mM. 2-morpholin-4-yl-6-thianthren-1-yl-pyran-4-one (KU55933, ATMi, Calbiochem) was dissolved in DMSO (Sigma-Aldrich) at 10 mM and was used at 10 μM final concentration. 7-hydroxystaurosporine (UCN-01, CHK1i, Calbiochem) was dissolved in DMSO at 100 μM and was used at 100 nM final concentration. The CHK2 inhibitor (CHK2 Inhibitor-II (BML-277), CHK2i, Calbiochem) was dissolved in DMSO at 1 mM and used at a final concentration of 400 nM. 8-(4-Dibenzothienyl)-2-(4-morpholinyl)-4H-1-benzopyran-4-one (NU7441, DNA-PKcsi, Tocris) was dissolved in DMSO at 10 mM and was used at 10 μM final concentration. 3-Amino-6-[4-(methylsulfonyl)phenyl]-N-phenyl-2-pyrazinecarboxamide (ATRi, VE-821, Haoyuan Chemexpress) was dissolved in DMSO at 10 mM concentration and was used at a 5 μM final concentration. Unless indicated otherwise, all inhibitors were added to the cells 1 h before irradiation and were maintained until collection for analysis. To induce the expression of ATR kinase-dead (ATRkd) protein that exerts a dominant-negative function on ATR, exponentially growing GM847-ATRkd cells were exposed for 48 h to doxycycline hyclate (DOX), (Sigma-Aldrich) at a concentration of 3 μg/ml. DOX was maintained in the cultures during the duration of the experiment.

### Indirect immunofluorescence (IF) and image analysis

For IF analysis^33^, cells were grown on coverslips coated with poly-L-lysine (Biochrom). S-phase cells were labelled with 10 μM of 5-ethynyl-2′-deoxyuridin (EdU) for 30 min. Further manipulations were as previously described^33^. Primary antibody for RPA70^64^ was diluted (1:300) in PBG solution. Alexa Fluor-conjugated secondary antibody, anti-mouse IgG Alexa Fluor 488 (ThermoFisher Scientific, A11001), was applied at 1:400 dilution for 1 h at RT. The EdU signal was developed using an EdU developing kit (ThermoFisher Scientific) according to the manufacturer’s instructions. Cells were counterstained with 100 ng/ml DAPI (ThermoFisher Scientific) at RT for 5 min and coverslips were mounted in PromoFluor antifade reagent (PromoCell). Scanning and analysis were carried out on an automated imaging system (Metasystems). For the quantification of parameters of interest, approximately, 1600 cells were analyzed to obtain ~200, EdU^+^, G_2_-phase cells.

### Flow cytometry analysis of DNA end-resection by RPA70 and BrdU signal quantification

DNA end-resection analysis using RPA70 detection were described previously^33^. Briefly, exponentially growing cells were pulse-labeled for 30 min with 10 μM EdU. When, BrdU detection was used, cells were grown for 24 h in presence of 10 μM BrdU and then labelled with EdU. EdU labeling was interrupted by rinsing the cells with pre-warmed PBS and fresh medium containing or not PIKK inhibitors was supplied. Cells were irradiated with indicated IR doses and were collected by trypsinization at the indicated times after irradiation. The unbound RPA was extracted by incubating in ice-cold PBS containing 0.2% Triton™ X-100 and cells were fixed in 3% PFA, 2% sucrose dissolved in PBS. Cells were blocked with PBG blocking buffer overnight at 4 °C and incubated with a monoclonal antibody raised against RPA70 (αSSB70B, mouse hybridoma cell line kindly provided by Dr. J. Hurwitz^64^) or in an anti-BrdU monoclonal antibody (Becton-Dickinson, 347580). Cells were washed twice with PBS and incubated for 1.5 h with a secondary antibody conjugated with AlexaFluor 488 (Invitrogen, A11001). EdU signal was developed using an EdU staining kit (ThermoFisher Scientific) according to the manufacturer’s instructions. Three-parameter analysis was carried out with a flow cytometer (Gallios, Beckman Coulter) and quantitated using the appropriate software as outlined in Fig. 5A (Kaluza 1.3, Beckman Coulter).

### Polyacrylamide gel electrophoresis (SDS-PAGE) and western blotting

SDS-PAGE and western blot analysis were carried out according to the previously published protocol^33^. Briefly, cells were collected by trypsinization and were washed in ice-cold PBS. Approximately 3 × 10^6^ cells were lysed in ice-cold RIPA buffer (ThermoFisher Scientific) supplemented with Halt^tm^ phosphatase and protease inhibitor cocktails (ThermoFisher Scientific), as recommended by the manufacturer and processed for SDS-PAGE and subsequently for western blotting as described^33^. The primary antibodies were: anti-RPA32 (mouse hybridoma cell line kindly provided by Dr. J. Hurwitz), anti-KU70 (529) (GeneTex, GTX77607), anti-ATR (Santa Cruz Biotechnology, sc-28901), anti-CHK1 (G-4) (Santa Cruz Biotechnology, sc-8408), anti-pCHK1-S345 (Cell Signaling Technology), anti-DNA-PKcs (Merck Millipore, PC127) and were used at 1:500 to 1:4000 dilutions. The secondary antibodies were anti-mouse IgG conjugated with IRDye680 or anti-rabbit-IgG conjugated with IRDye800 (Li-COR Biosciences, 92668020 and 92632211) at 1:15,000 dilution. Immunoblots were visualized by scanning the membranes in an Odyssey infrared scanner (Li-COR Biosciences). Digital images were processed using the brightness and contrast functions of the dedicated Odyssey software. Raw non-cropped scanned membranes are presented on Fig. S4A–C.

## Supplementary information

Supplementary information
