# Supplementary material for: DNA-PKcs and ATM epistatically suppress DNA end resection and hyperactivation of ATR-dependent G2-checkpoint in S-phase irradiated cells
Source: Sci Rep. 2019 Oct 10;9:14597. doi: 10.1038/s41598-019-51071-6 (PMC6787047; doi:10.1038/s41598-019-51071-6)
Supplement: Supplementary file 1 — Supplementary information [file 41598_2019_51071_MOESM1_ESM.pdf]

Supplementary Information

DNA-PKcs and ATM epistatically suppress DNA end resection and hyperactivation of ATR-dependent G_2-checkpoint in S-phase irradiated cells

Emil Mladenov, Xiaoxiang Fan, Katja Paul-Konietzko, Aashish Soni and George Iliakis

Supplementary Figure Legends

Figure S1: (A and B) Representative plots showing the regions used for gating the BrdU positive, G_2-phase, A549 cells used to determine the signal plotted in Figure 1C. Upper panel: Cell cycle distribution determined by analyzing PI signal. Lower panel: Contour plots of BrdU *versus* PI signal used to define the gates for BrdU positive cells. **(C and D)** As in Figure S1A and S1B for AT5Biva cells. **(E)** Percentage of G_2-phase, AT hTert cells exposed to 10 Gy of IR in the presence or absence of ATRi.

Figure S2: (A) As in Figure 1A for DNA-PKcs deficient, HCT116 cells (HCT116$^{DNA\text{-}PKcs-/-}$) exposed to 2 Gy of IR and treated with the indicated inhibitors. **(B)** As in Figure S2A for DNA-PKcs proficient HCT116 cells. **(C)** As in Figure S2A for A549 cells. **(D)** As in Figure S2A for DNA-PKcs deficient, HCT116 cells exposed to 10 Gy of IR and treated with the indicated inhibitors. **(E)** Left panels show the cell cycle distribution of S-phase enriched, parental A549 cells (A549^{wt}), at the time of exposure to 10 Gy IR (0 h), as well as 9 h after IR exposure. Right panels show similar results for a CRISPR/cas9 generated DNA-PKcs deficient A549 cells (A549$^{DNA\text{-}PKcs-/-}$). **(F)** As in Figure 2SE for AT hTert cells.

Figure S3: (A) Quantitative analysis of DNA end resection using median values of RPA70 signal intensity. Other details as in Figure 5C (lower panels). **(B)** RPA70 signal intensity in EdU$^+$, G_2-phase, A549 cells at 3 and 6 h after exposure of S-phase cells (EdU$^+$) to 10 Gy X-rays, in the presence or absence of ATRi. **(C)** Quantitative analysis of DNA end resection using median values of BrdU signal intensity. Other details as in Figure 6D. **(D)** As in Figure S3B for AT hTert cells exposed to 10 Gy and analyzed at 3, 6, and 9 h post-irradiation in the presence or absence of ATRi. **(E)** RPA70 signal intensity in EdU$^+$, G_2-phase, HCT116 cells, lacking DNA-PKcs activity, treated with indicated inhibitors and irradiated with 10 Gy. **(F)**

RPA70 signal intensity in EdU^+, G_2-phase, M059J cells 6 h after exposure of S-phase cells (EdU^+) to 10 Gy X-rays, in the presence or absence of CHK1i.

Figure S4: (A) Raw western blot data of experiments shown in Figure 1D. **(B)** Raw western blot data of experiments shown in Figure 3E. **(C)** Raw western blot data of experiments shown in Figure 3F.

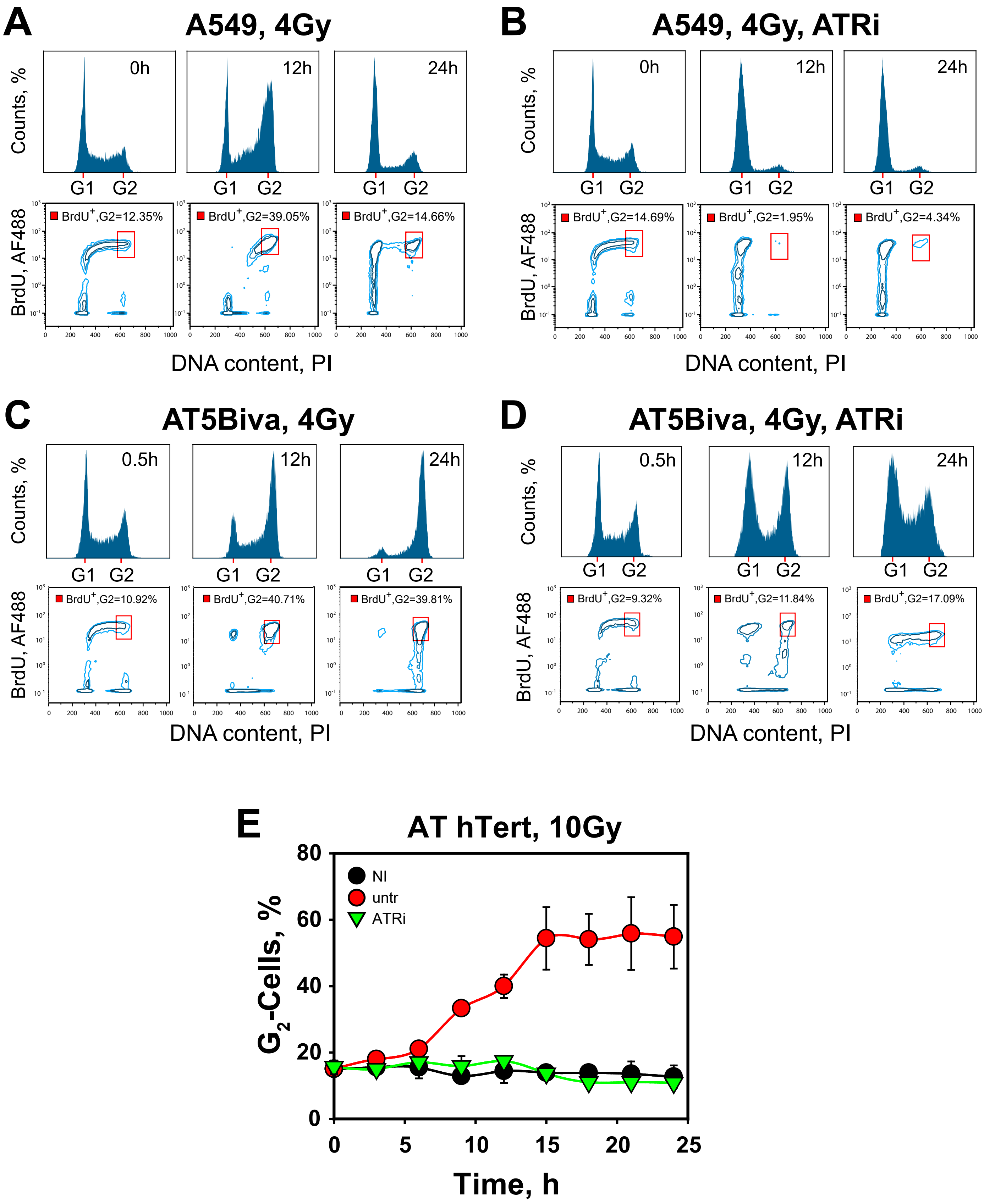
A
A549, 4Gy
0h
12h
24h
Counts, %
G1 G2
BrdU+,G2=12.35%
BrdU+,G2=39.05%
BrdU+,G2=14.66%
BrdU, AF488
DNA content, PI
B
A549, 4Gy, ATRi
BrdU+,G2=14.69%
BrdU+,G2=1.95%
BrdU+,G2=4.34%
C
AT5Biva, 4Gy
0.5h
BrdU+,G2=10.92%
BrdU+,G2=40.71%
BrdU+,G2=39.81%
D
AT5Biva, 4Gy, ATRi
BrdU+,G2=9.32%
BrdU+,G2=11.84%
BrdU+,G2=17.09%
E
AT hTert, 10Gy
NI
untr
ATRi
G2-Cells, %
Time, h

Figure S1

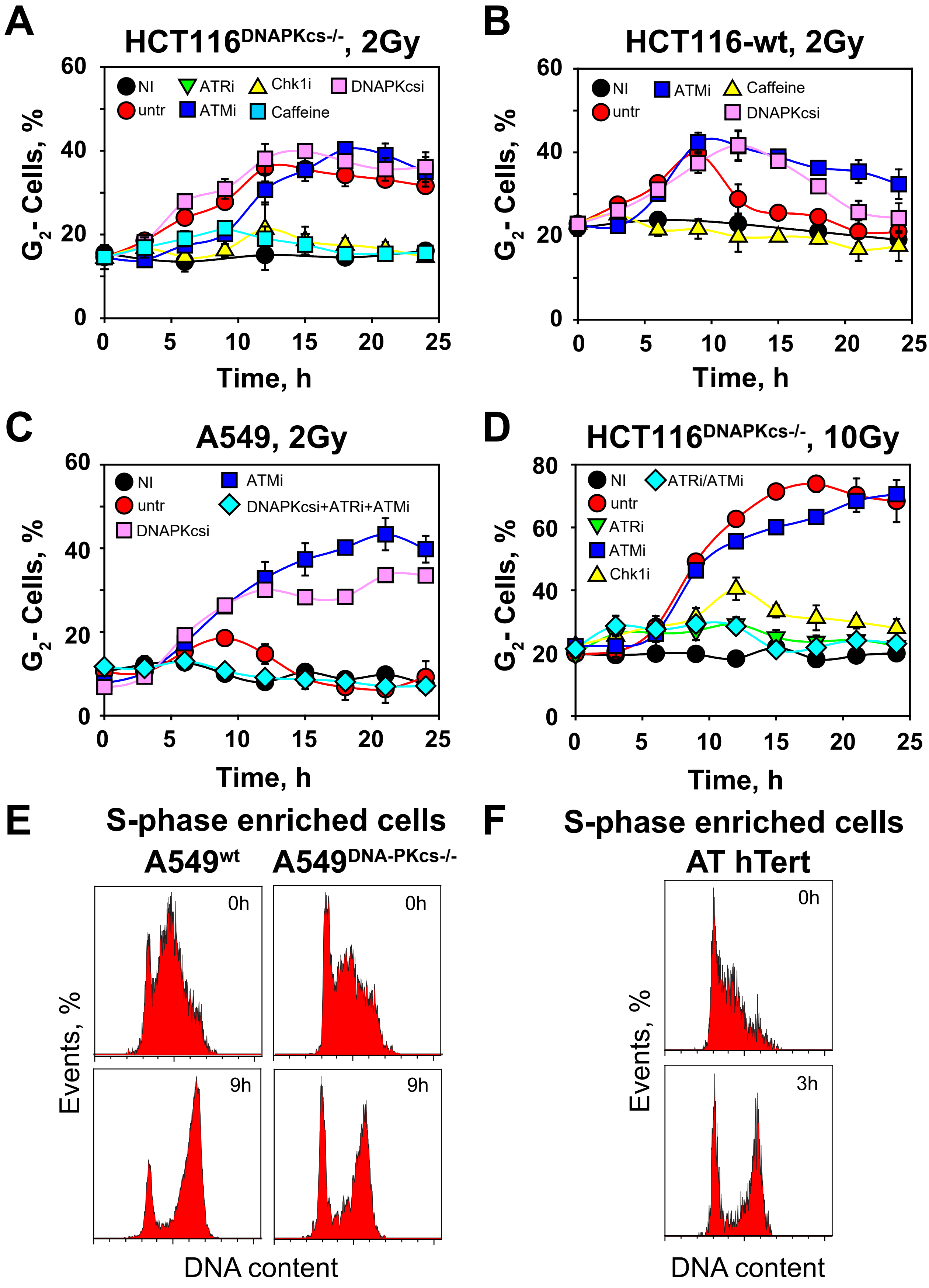
A
HCT116DNAPKcs-/-, 2Gy
NI
untr
ATRi
ATMi
Chk1i
Caffeine
DNAPKcsi
G2- Cells, %
Time, h
B
HCT116-wt, 2Gy
NI
untr
ATMi
Caffeine
DNAPKcsi
G2- Cells, %
Time, h
C
A549, 2Gy
NI
untr
DNAPKcsi
ATMi
DNAPKcsi+ATRi+ATMi
G2- Cells, %
Time, h
D
HCT116DNAPKcs-/-, 10Gy
NI
untr
ATRi
ATMi
Chk1i
ATRi/ATMi
G2- Cells, %
Time, h
E
S-phase enriched cells
A549wt
A549DNA-PKcs-/-
0h
9h
Events, %
DNA content
F
S-phase enriched cells
AT hTert
0h
3h
Events, %
DNA content

Figure S2

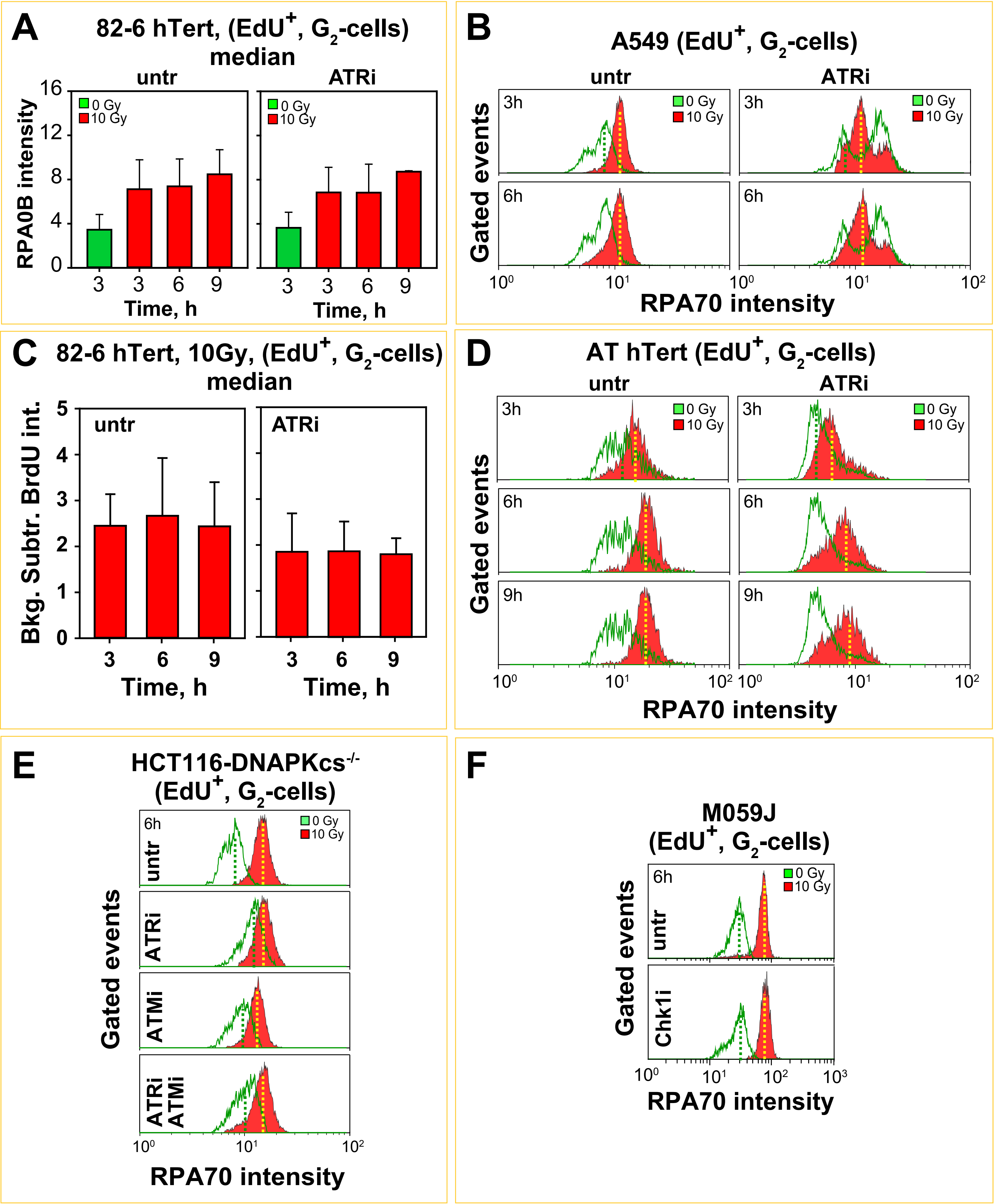
A
82-6 hTert, (EdU+, G2-cells)
median
untr
ATRi
RPA0B intensity
0 Gy
10 Gy
Time, h
B
A549 (EdU+, G2-cells)
untr
ATRi
Gated events
3h
6h
RPA70 intensity
C
82-6 hTert, 10Gy, (EdU+, G2-cells)
median
Bkg. Subtr. BrdU int.
untr
ATRi
Time, h
D
AT hTert (EdU+, G2-cells)
untr
ATRi
Gated events
3h
6h
9h
RPA70 intensity
E
HCT116-DNAPKcs-/-
(EdU+, G2-cells)
Gated events
6h
untr
ATRi
ATMi
ATRi ATMi
RPA70 intensity
F
M059J
(EdU+, G2-cells)
Gated events
6h
untr
Chk1i
RPA70 intensity

Figure S3

GM847-ATRkd

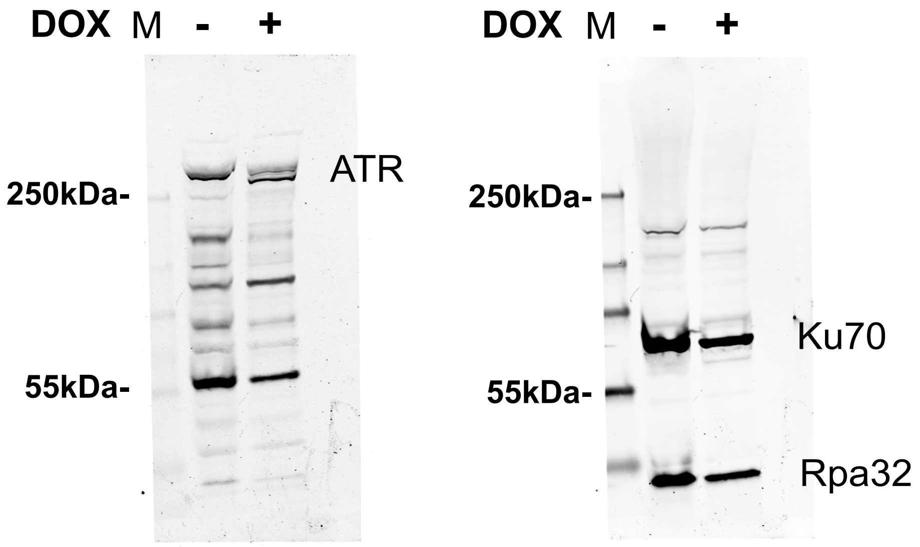

Figure 1D

Figure S4A

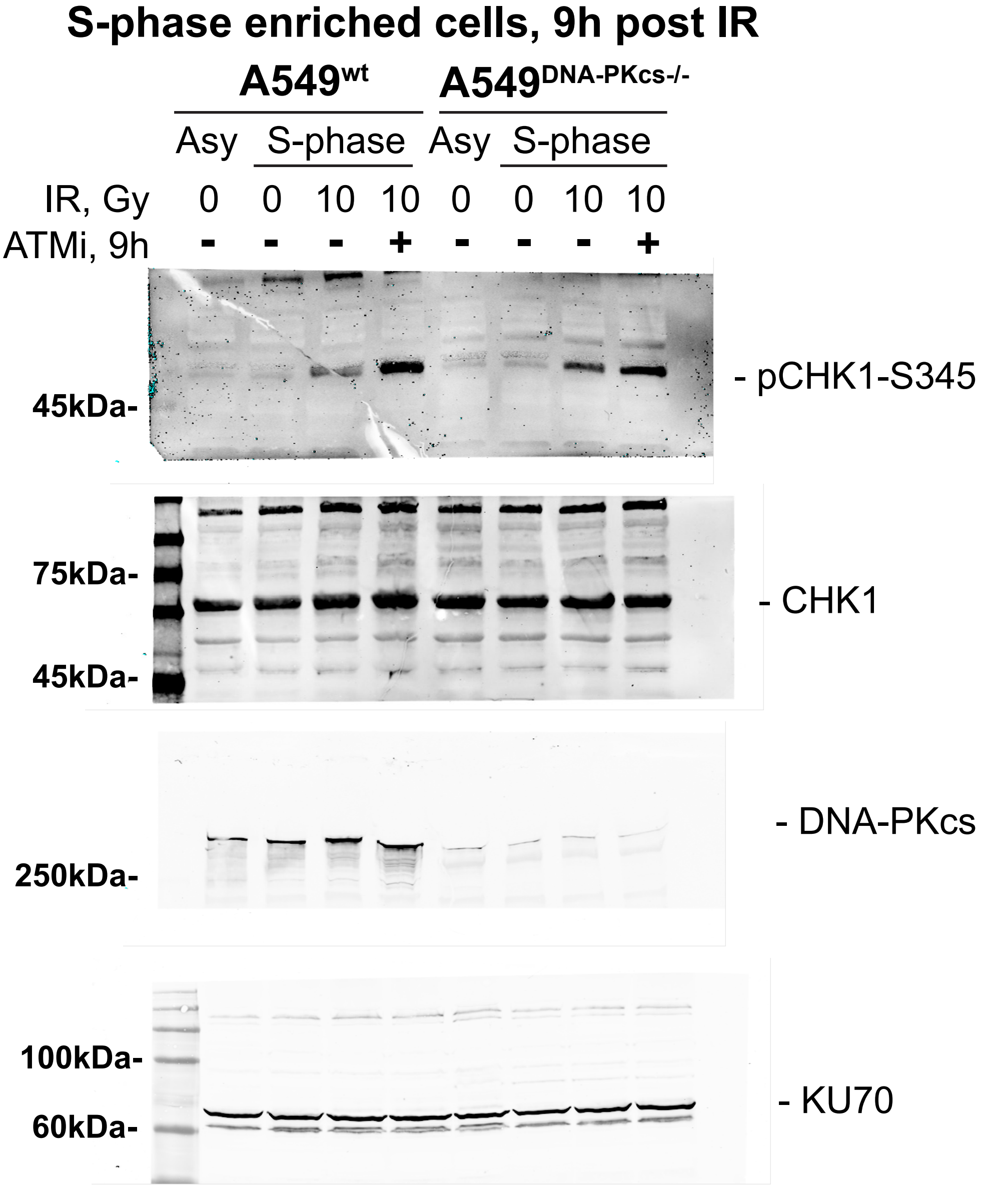

Figure 3E

Figure S4B

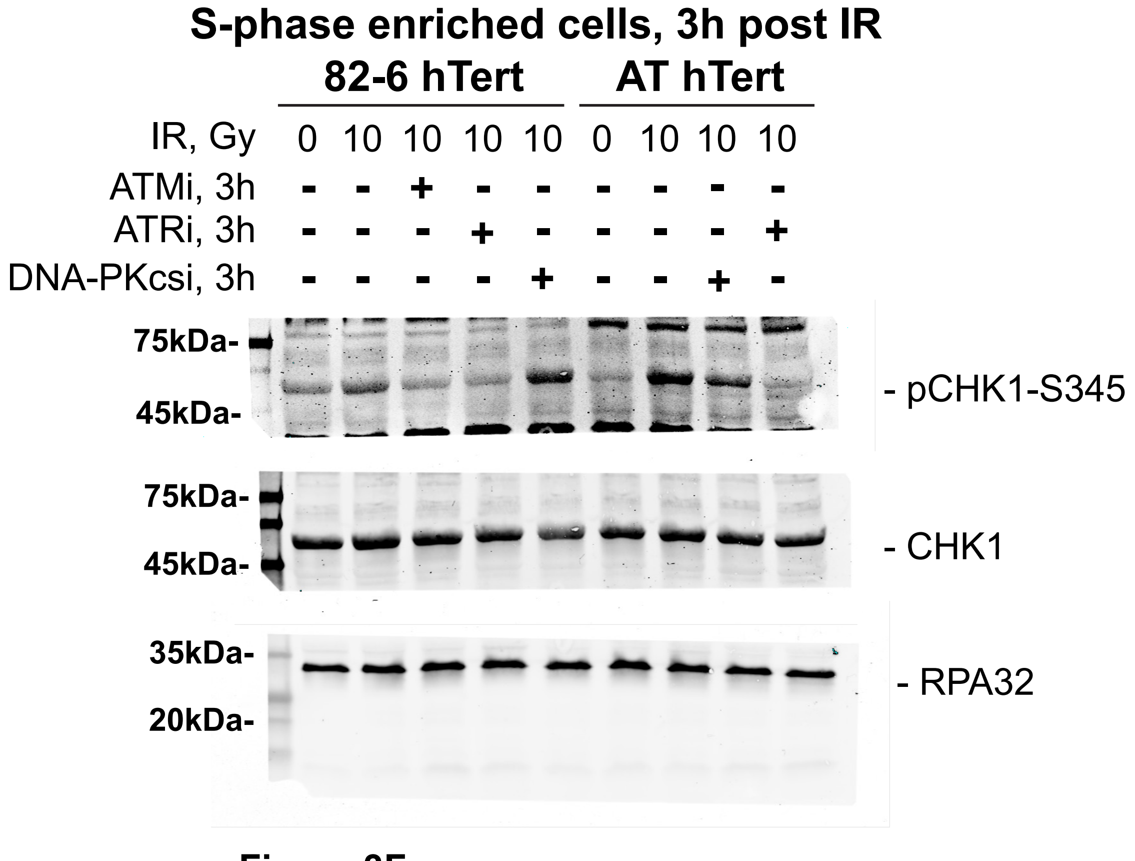

Figure 3F

Figure S4C
